# Effect of a Job Demand-Control-Social Support Model on Accounting Professionals’ Health Perception

**DOI:** 10.3390/ijerph15112437

**Published:** 2018-11-01

**Authors:** José Joaquín Del Pozo-Antúnez, Antonio Ariza-Montes, Francisco Fernández-Navarro, Horacio Molina-Sánchez

**Affiliations:** 1 Financial Economic and Accounting Department, Universidad Loyola Andalucía, 14004 Córdoba, Spain; jjpozo@uloyola.es (J.J.D.P.-A.); hmolina@uloyola.es (H.M.-S.); 2 Management Department, Universidad Loyola Andalucía, 14004 Córdoba, Spain; 3 Department of Business Administration, Universidad Autónoma de Chile, 7500912 Santiago, Chile; 4 Quantitative Methods Department, Universidad Loyola Andalucía, 14004 Córdoba, Spain; fafernandez@uloyola.es

**Keywords:** Perceived Occupational Health (POH), Job Demands-Control-Social Support (JD-R) model, professional accountants

## Abstract

The Job Demand-Control and Job Demand-Control-Support (JDCS) models constitute the theoretical approaches used to analyze the relationship between the characteristics of labor and occupational health. Few studies have investigated the main effects and multiplicative model in relation to the perceived occupational health of professional accountants. Accountants are subject to various types of pressure in performing their work; this pressure influences their health and, ultimately, their ability to perform a job well. The objective of this study is to investigate the effects of job demands on the occupational health of 739 accountants, as well as the role of the moderator that internal resources (locus of control) and external resources (social support) have in occupational health. The proposed hypotheses are tested by applying different models of neural networks using the algorithm of the Extreme Learning Machine. The results confirm the relationship between certain stress factors that affect the health of the accountants, as well as the direct effect that the recognition of superiors in occupational health has. Additionally, the results highlight the moderating effect of professional development and the support of superiors on the job’s demands.

## 1. Introduction

As indicated by Reference [1], workplace health management is crucial for improvements in psychosocial working conditions and health. Promoting healthy work environments is a matter of ethics as well as business interest, since the most competitive companies are those with mentally and physically healthy workers due to policies supporting and protecting their health [2]. There is no doubt that work is part of the social dimension of health. The National Institute for Occupational Safety and Health (NIOSH) recognizes the importance of the well-being of workers, their families and communities through a series of factors linked to the employment relationship, such as wages, hours of work, workload and stress levels, interactions with co-workers and supervisors, access to paid leave, and health-promoting workplaces [3]. Therefore, Total Worker Health promotes an integral intervention in health where measures aimed at the protection of the health of employees are combined with others supportive of well-being. From this perspective, Total Worker Health integrates the individual dimension of health with the organizational dimension and the environment [4]. The Job Demand-Control (JDC) model [5] and the Job Demand-Control-Support (JDCS) model [6,7] constitute the most widely used theoretical approaches to understanding and interpreting the relationships between the characteristics of health, work and well-being [8]. In fact, according to Reference [9], the occupational stress literature is dominated by these models.

Undoubtedly, every effort to improve the integral health of workers translates into greater working efficiency, as shown by the meta-analysis by Reference [10]. The JDCS model warns that the greatest risks to physical and mental health are manifested among workers who experience a high isolation-strain (iso-strain) job—that is, those that are subject to high job demands in a context of low control or decision latitude and low social support (iso-strain hypothesis). However, empirical evidence of this three-way interaction effect is still limited, primarily by the variety that exists in terms of the characteristics and conditions of labor between different jobs and occupations. Many authors, such as Reference [11], indicate that the samples used to test the JDCS model should be as homogeneous as possible, although they are heterogeneous with respect to the level of exposure of workers to the labor environment variables. This circumstance suggests the need to carry out research in occupations with similar characteristics. In addition, not all professions are subject to the same degree of strain, but some occupations tend to combine certain conditions that make workers in those jobs more vulnerable in terms of their physical and mental health [12].

Accountants play an important role in the financial market because they provide accurate information for decision making. Expert judgement and mental equilibrium are required for sound decisions. Despite this, an important gap of research exists in relation to pressures on accountants and its effects. Recently, a professional publication has shown how leading firms of accountants have taken steps to improve their health at work: Training the team leaders on issues of occupational health, creating spaces for healthier work practices, knowledge of such risks, sharing experiences among team members, etc. [13].

A profession as important as accounting is subject to heavy job demands that may affect one’s perceived occupational health (POH). Occupational health is the result of the confluence of a number of stress factors and mechanism moderators in organizations. The effect of stressors on accountants has been studied in terms of different effects: For example, dysfunctional behaviour [14,15,16], personal well-being [17,18], labor satisfaction [17], performance [17] or turnover intention [17,19,20,21,22]. This research used theoretical frameworks compatible with those that explain the effect of the job demands in occupational health deterioration and, in particular, one of its major manifestations: Burnout [17,19,20,21,22,23,24,25,26]. Thus, the literature on the accountancy profession has identified several main categories of stressors, which highlight role overload, role conflict and role ambiguity [23]. The Job Demand-Job Control-Social Support (JDCS) model provides a holistic framework to investigate the direct effects and moderators of these stressors on occupational health. To our knowledge, this model has not has been tested on a group of accountants, so this paper covers an important gap in the literature.

This paper tries to cover gaps in the research on this topic in the European context. Thus, the objective of this study is to investigate the effects of job demands on the occupational health of the accountants, as well as the role of the moderator that internal resources (locus of control) and external resources (social support) have in occupational health. To meet the objectives of this research, Section 2 presents the theoretical framework and hypotheses that are derived from the framework. Section 3 describes the empirical study design and methodology used, which are based mainly on the analysis of neural networks. Section 4 displays the main results. The article ends with a discussion of the results (Section 5) and the main limitations of the study and future lines of research (Section 6).

## 2. Theoretical Framework and Hypothesis Development

This work deals with the study of the health of accountants based on a theoretical framework that uses the Job Demand-Control-Support (JDCS) model formulated by References [5,6,7]. Different authors have used this model to explain the effect of the job demands on occupational health [27,28,29]. 

**Figure 1:**
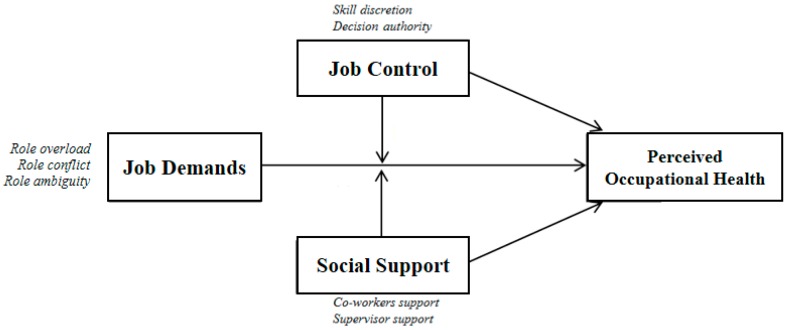
Model of Job Demand-Control-Support (JDCS) applied to the work of accountants.

The general formulation of the JDCS model states that job demands cause a strain. However, it may moderate (or intensify) depending on the degree of control that the employee has on their work and the social support available (see Figure 1). 

Job control involves the employees’ ability to organize their work and adopt their own initiatives. This perspective would have to be considered a double dimension of the work. On the one hand, the axis of the strain warns that jobs with high demands and low autonomy generate more strain, as opposed to jobs with low demands and high control, where the level of strain would be small. On the other hand, the axis of learning suggests that there is a type of challenging job with a favorable environment for career development when demands are high but also has a high degree of autonomy and implementation of their skills, as is possibly the case of this research.

The model proposed by Reference [5] originally contains two hypotheses. First, the strain hypothesis suggests that demanding activities with low control increase the risk of worker well-being, which is the effect of an additive and multiplicative character. Second, the buffer hypothesis emphasizes a character moderator that exerts job control on the relationship of job demand-strain. According to Reference [30], the two scenarios are not alternatives but are complementary, resulting in an extension of the hypothesis of the voltage buffer hypothesis. 

As indicated above, in an extension of the original model, Reference [6] incorporated a second buffer factor of the job demands: Social support for both co-workers and supervisors. Given that the nature of the work of the accountants is based on teamwork, the dimension of social support in this context is a particularly relevant research framework.

### 2.1. Job Demands of Accountants

Accountancy firms are hierarchical and competitive entities to the point that several authors, such as Reference [31], believed that stress is a tool used intentionally by managers to achieve the maximum effectiveness from their subordinates in accounting firms. For example, the pressure of time improves the effectiveness of professional accountants, as Reference [32] observes, especially in the decision-making process, since it causes them to focus only on relevant information. However, work overload and time pressures, far from improving the performance of the professionals, endanger the quality of the work. The duality of effects, positive and negative, that the scientific literature has identified in relation to work overload is explained with the approach of the Arousal Theory, for which the relationship between stress and performance is U-shaped inverted, as pointed out in Reference [33]. As Reference [34] points out, the elimination of stress in accounting is a utopia, given the characteristics of this type of activity—for instance, seasonality linked to compliance with trade regulations, tight deadlines (so that customers can give timely information to the markets), tight time and monetary budgets by the increased competitiveness of the sector, the complexity of some decisions of the audit, manipulation of information by customers. However, scientific research can provide useful tools that contribute to coping and managing stress better.

The Stress Diagnosis Survey (SDS) of Reference [35] is one of the most used tools to analyze the demands that cause greater stress to the accountants. The SDS considers two categories of stressors: Individual and organizational. Individual stress includes role conflict, role ambiguity, quantitative and qualitative overload, time pressures, responsibility, professional careers and the scope of the work. Organizational factors include internal policy, development of human resources, compensation policy, participation, underutilization, style of supervision and organizational structure [36,37,38]. 

The model of Reference [23] is a reference for some work done later in the accounting field [17,21,24]. Nevertheless, as is noted above, perhaps the theoretical framework that is more accepted to explain the influence of the employment context in the well-being of employees is the JDCS model. However, in the specific field of audit accounting, its use has been much more limited. Thus, the relationship between stressors and the quality of the work of audit under this framework is explained by Reference [39], noting that the quality of the audit work is not affected by stressful situations if it remains under control. These authors warn that the buffering effect does not occur in the first year in which a client is audited. This will manifest in successive years as experience is acquired with the customer since personal competencies are enhanced to address work that translates into less stress.

This research uses the classification of the job demands that Reference [23] discusses around three broad categories of stressors: Work overload, role conflict and role ambiguity.

#### 2.1.1. Work Overload

Work overload is observed the factor that most influences the deterioration of occupational health [38]. This is produced by taking on a large number of engagements, the tight time allowance to carry out the work or by the imposition of overly tight deadlines. If the prestige of the accountant increases, the demand for their services increases too, resulting in the paradox that excess demand on actual capacity may harm the work quality [40].

Another troubling circumstance is that increasing competition forces price adjustment and reduces the budgets of the implementation of the engagements. This can lead to two dysfunctional practices: Reduce the scope of procedures, again compromising the quality of the work; or underreporting time [41,42,43]. Time pressure inversely affects the quality of work. Performing audit judgements that entail a high level of subjectivity means that the professional accountant may be tempted to relieve pressure by skewing his own judgement. In this way, professional accountants who work with more time pressure assessed a lower risk of significant error in the audited financial statements, which leads them to decrease the intensity of the procedures and the workload [16,44]. In addition, reporting less than the actual number of hours worked (time underreporting) produces a double negative effect. On the one hand, planning future engagements considers the budget timetable of the previous year, thus conditioning the planning of future work. On the other hand, the evaluation of a professional’s performance is distorted and is expected within an unrealistic time frame. Underreporting time is considered a more ethical strategy than devoting fewer hours than necessary when the budget is too tight [45].

Work overload also occurs when deadlines are so tight that they cause negative consequences for the quality of the work [46,47]. For example, professional accountants tended to consider less important errors detected on tight deadlines, especially when these deadlines have been established by the own professional accountants [48]. The obligation to comply with the rule of law creates peaks and concentration of work, which result in work overload and higher role conflict, which leads to emotional exhaustion among professional accountants, as References [25,26] have shown.

#### 2.1.2. Role Conflict

Economic effects arising from the financial information prepared by accountants can sometimes lead to conflicts of an ethical nature. For example, the auditors attempt to preserve relations with customers. Therefore, using findings that may have a negative influence on the client, their judgements tend to be more relaxed, especially if the work contains a high level of subjectivity (for example, judgements on the materiality of the deviations identified in the internal control [48]). Thus, as the client portfolio of the professional accountant expands, the independence level increases, thus reducing the role conflict but increasing the workload [49].

Role conflict is more likely to occur at lower levels of a professional career due to the pressure from superiors (managers and partners) on these individuals, which has come to be called “pressure by obedience”. This pressure may cause less-skilled team members to violate professional standards to meet the demands of their superiors [50].

All of the above issues translate into greater role conflict, which, according to Reference [34], cause greater stress and lower job satisfaction, thus affecting the health of the accountants.

#### 2.1.3. Role Ambiguity

Another factor that can affect the quality of the work of professional accountants is the lack of understanding of the tasks to be carried out [15]. As indicated by Reference [31], the risk of significant errors during the performance of work creates a sense of fear among professional accountants. This sensation may have both positive (e.g., stimulates professional diligence) and negative effects (since it can induce the adoption of defensive strategies). What does appear clear is that the accountants experience less comfort to perform more complex tasks than when they perform more routine tasks [51].

Undoubtedly, as Reference [34] proposed, all these elements of role ambiguity have a negative influence on satisfaction and perception of performance.

### 2.2. Job Control

The negative effects that generate the high job demands can be buffered or intensified depending on the degree of control that the accountant has on the activity. This control is highlighted mainly by two factors: The possibility of applying one’s own skills (skill discretion) and the level of autonomy over decisions that affect you (decision authority) [5]. In a recent study, professionals with greater competence and autonomy experienced less role ambiguity [52]. This circumstance occurs because higher levels of competition allowed attention to the complexity associated with the tasks of the profession with less pressure. At the same time, these authors found that less autonomy reduced access to information, which resulted in greater ambiguity.

There is evidence that shows that the degree of job autonomy in decision-making, such as the ability to decide one’s workload, moderates some variables related to occupational health, such as stress [53]. Although the work overload may cause adverse effects on the quality of the work, the fact is that accountancy firms are impregnated with an organizational culture that accepts, encourages and imposes high standards of work that translate into levels of high demand. Despite this, there is empirical evidence showing that if the accountant chooses the workload voluntarily, harmful effects on the quality of the work are not produced.

Job autonomy in the field of auditing can cause dysfunctional behaviours; in fact, in a sample of Chinese professional accountants, the dysfunctional behaviour increased because the professional had greater autonomy [54]. In a positive sense, these authors also noted that accountants perceived job autonomy as a sign of support from the organization, which resulted in greater job satisfaction. 

The pressure caused by tight time budgets also leads to dysfunctional behaviours. However, these vary at different hierarchical levels and are more likely in positions requiring less experience—for example, in roles with less autonomy in the activity planning [46]. In addition, these authors observed that the perception of a greater degree of involvement in time programming has a positive influence on the achievement of the budgetary targets.

On the other hand, control over the activity also manifests itself when accountants can fully display their abilities. This situation makes work exciting and encourages accountants to do their best work rather than adopting dysfunctional behaviours [47]. In this way, because the content of the tasks corresponds to the level of professional development, stressors are cushioned by the incentive that involves applying one’s own professional competencies. Professional judgement develops as experience is gained. In the early professional stages, tasks are more structured and require a level of minor professional judgement. Over time, the responsibility of professional accountants on more complex decisions leads to higher ambiguity. Without a doubt, the experience provides greater comfort in complex decision-making, in keeping with the principles of Social Cognitive Theory. The experience leads to better assessments of the risk of significant errors [44,55], which determines greater control of the activities and leads to highly stimulating work when personal skills are applied to help solve complex situations. Finally, because experience is gained when a customer increases knowledge about such situations [56,57], improving control over activities and diluting the negative effects of work overload may result in the quality of the work of the professional accountant [58].

### 2.3. Social Support

Several authors believe that quality relations between the accountancy firms and their professionals develop more intensely when they perceived a fair deal and when they feel supported by the organization, which reduces burnout and intention to leave the organization [19,22].

Collectivism exerts a positive influence on the level of well-being of professional accountants, which is measured by its three components: Job satisfaction, work-life balance and life satisfaction [18]. For these authors, as the engagements become more complex, professional accountants develop a feeling of belonging to the work and a spirit of team-oriented organization. In this context, the behaviour of superiors is influential, since they create an organizational culture, which defines what practices are encouraged and desired by the firm. Thus, if superiors expressly and honestly reject the practices that generate stress (such as the underreporting the hours spent in completing work), subordinates feel that their stress levels are alleviated. 

In this sense, there is a negative relationship between job autonomy and counterproductive behaviours, an effect that is compensated for and reversed by a set of factors, among which perceived organizational support stands out [54]. This circumstance suggests that the negative consequences associated with job autonomy are mitigated in organizations that promote positive attitudes among their employees. In addition, firms that promote values and ethical behaviour increase the level of socialization of its professionals, significantly reducing dysfunctional practices that could arise from time pressures [45].

Social support depends greatly on one’s superiors. The style of leadership of these superiors has a direct effect on the performance of teams and in superior-subordinate relations, even in relations between the members of the team. Team performance improves when superiors stimulate innovation, serve the personal needs of team members, offer positive reinforcement and conform to the budgets schedules [59]. More recently, in an investigation on accountants, leaders who promote a strong team culture achieve smoother communication and greater cohesion among members [60].

Feelings emerge in opposite directions when subordinates do not feel supported by their immediate supervisor. This is something that happens all too often in accountancy firms due to the feeling of fear that is cultivated more or less informally with the intention of stimulating monitoring, promoting self-improvement, mitigating the anaesthetizing effect of habit and maintaining reputation [31]. This feeling is more unusual because auditors are accountable in more instances and when more complex tasks become especially intense [51].

### 2.4. Hypothesis Development

**Figure 2:**
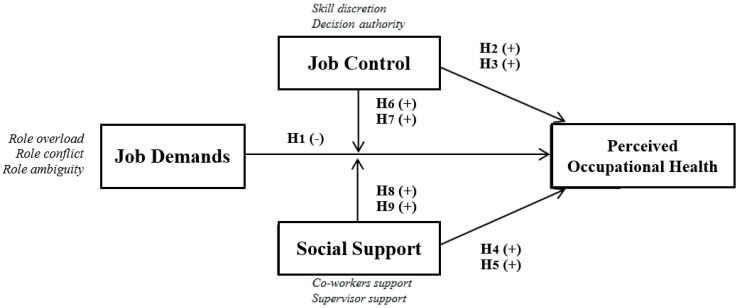
Model hypotheses.

Figure 2 shows the theoretical model on which this work is based, as well as the hypotheses that seek to demonstrate the purpose of this investigation. These are divided into two groups: The direct effects of three model factors and moderator effects.

#### 2.4.1. Direct Effects

**Hypothesis 1** **(H1).** *As the job demands become more demanding, perceived occupational health of professional accountants decreases.*

**Hypothesis 2** **(H2).** *As accountants have greater autonomy in the implementation of their professional skills, perceived occupational health increases.*

**Hypothesis 3** **(H3).** *As the accountant has higher degree of autonomy in decision-making, perceived health increases.*

**Hypothesis 4** **(H4).** *As the accountant has more support from his superiors, perceived occupational health increases.*

**Hypothesis 5** **(H5).** *As the accountant has greater support from peers, perceived occupational health increases.*

#### 2.4.2. Moderating Effects

**Hypothesis 6** **(H6).** *Autonomy in the implementation of professional competences buffers the relationship between job demands and perceived occupational health.*

**Hypothesis 7** **(H7).** *Autonomy in decision-making buffers the relationship between job demands and perceived occupational health.*

**Hypothesis 8** **(H8).** *Support from one’s superiors serves as a moderator of the relationship between the demands of the job role in perceived occupational health.*

**Hypothesis 9** **(H9).** *Peer support serves as a moderator of the relationship between the demands of work on perceived occupational health.*

## 3. Design of the Empirical Research

### 3.1. Sample

The data used for the development of this research were obtained from the sixth European Working Conditions Survey, which was developed by the European Foundation for the Improvement of the Conditions of Life and Work in 2015 [61]. This survey analyzes the working conditions in the 27 countries of the European Union, providing valuable information on different aspects of the working conditions in Europe: Attitudes, perceptions and behavior of employees. The population consists of all persons aged 15 or above who are employed or self-employed and whose usual place of residence is in one of the member states of the European Union. The field work was carried out in 2015 based on 43,850 valid surveys.

To achieve the objectives of the present research, a sub-sample of 739 professional accountants were extracted using codes 2411 (accountants) and 3313 (accounting associate professionals) of the International Standard Classification of Occupations. 

Of the respondents, 75.2% are women, while the remaining 24.8% are men. The average age of the professional accountants is 43.9 years. Most respondents working in the private sector (82.5%) with a contract mainly have indefinite work contracts (88.8%) that are an average of 10.9 years old. With respect to the main variable of this research, it should be noted that 17.7% of respondents believe that the development of the work directly affects their health, compared to 82.3% who considered otherwise.

### 3.2. Measures

The dependent variable of this research is the perceived occupational health of professional accountants. A single item measures this variable: Whether the professionals perceive that their work directly affects their health. The respondents had three response options: No; yes, mainly positively; and yes, mainly negatively. 

The job demands were measured using 7 items grouped into three categories: Role overload (e.g., “Does your job involve working with tight deadlines?”), role ambiguity (e.g., “Do you know what is expected of you at work?”) and role conflict (e.g., “Does your job involve being in situations that are emotionally disturbing for you?”). 

The scale of job control integrates 4 items that measure skill discretion (e.g., “Does your job involve solving unforeseen problems on your own?”) and 7 items that assess the decision authority (e.g., “Are you consulted before objectives are set for your work?” or “Do you have a say in the choice of your work colleagues?”).

Finally, the scale of social support measures support from superiors with 7 items (e.g., “Your immediate supervisor provides useful feedback on your work” or “Your immediate boss encourages and supports your development”), while the support of co-workers is determined by a unique item that ask directly if “your colleagues help and support you.” 

### 3.3. Methodology

The methodology proposed in this research is developed in three phases: Preparation of the constructs of the first order from the application of a factor analysis, analysis of neural networks with the Extreme Learning Machine algorithm, and finally, interpretation of the resulting model using a sensitivity analysis.

A factor analysis was run with a rotation promax on the original data set. Promax rotation allows the factors obtained to be correlated (unlike the varimax rotation or orthogonal rotation). Following the recommendations of Reference [62], items that were not correlated with any specific factor were excluded from the analysis, while the loading used for other factor was 0.40. Variables that are grouped without any logical meaning according to the nature of the problem were also eliminated. The factor analysis revealed the existence of first-order factors for the constructs: Job Demands (JD), Skill Discretion (SD), Decision Authority (DA) and Supervisor Support (SS). The construct Co-Worker Support (WS) was composed of a single item. Since the input variables were represented in different ranges, it was decided to standardize them to a [0, 1] scale linearly according to the function min (max). 

The main analysis was carried out with artificial neural networks. This method has shown satisfactory results in solving complex problems and constitutes a useful tool in data analysis of different areas or disciplines: Medicine, economics, engineering, biology and psychology [63]. Increasingly, more authors appreciate their applicability with regard to models derived from classic statistics [64,65]. From a methodological perspective, the priority themes that apply neural networks deal with the classification of patterns (classification and prediction) and approximation of functions [63]. It is possible that the growing interest in neural networks lies in its capacity for the treatment of nonlinear problems [66], since better yields are achieved because there is independence from the fulfilment of the theoretical assumptions of traditional techniques. Neural networks have proven to be an effective tool for classifying cases under the non-linearity hypothesis. A neural network is a linear model in which the basis functions can be a sigmoid type. In the case that concerns us, the analysis was performed using a neural network in a single layer, which allows for modelling interactions of an order greater than two (and not only multiplicative); interactions will be key to analyzing the moderator effect of job control and social support. The parameters of the model have been estimated using the algorithm Extreme Learning Machine [67]. In this algorithm, the weights of the input layer to the hidden layer (which models the nonlinear part of the system) are initialized randomly. In addition, the parameters that bind the hidden layer with the layer’s output are estimated analytically after solving a problem of least squares with regularization. 

To finish the investigation, a sensitivity analysis was conducted. The main disadvantage of the models of neural networks is that they are considered a “black box” type, since they are effective at finding hidden relationships between inputs and outputs with a high capacity for approximation, but they do not provide information on how they have managed to do so. This limitation causes many academics to scrap the use of these models in their research. A sensitivity analysis is used to overcome this restriction. The present study uses a global sensitivity analysis inspired by a decomposition functional ANOVA [68]. This method makes it possible to decompose the nonlinear function on a set of elements associated with the parts of the independent variables, the interactions of the variable two by two, to the interactions between variables three to three and so on until all interactions of the input variables are analyzed. This methodology was already proposed for the classification problem and has been adapted ad hoc in this study for the case of regression [69]. To evaluate the stability of the method, an analysis with two subsamples that gave rise to two estimates of parameters of sensitivity (estimate 1 and 2 estimate) was performed. 

## 4. Results

In the case of factor analysis, the construct Job Demands (JD) was reduced to a single component that explained 35.38% of total variance (the other elements presented eigenvalues below one). The construct Job Control (JC) was composed of two elements: Skill Discretion (SD) and Decision Authority (DA). These are summarized in two factors that explained 38.55% and 46.84% of variance, respectively. The construct Social Support (SS) was composed, as explained above, by two elements: Supervisor Support (SS) and Co-Worker Support (WS). The first is represented by a factor that explained 75.54% of variance, while the second was a construct consisting of a single item.

First, we analyze the mean square error (MSE) of the linear regression, including interactions considered in this research, compared to the model of the trained neural network according to Extreme Learning Machine. The classical model earned an MSE of 31.8862 and a neural network 11.4588 MSE, which justifies that the neural network model summarizes data more effectively than the classic model. The result of the regularization was cross-validated, resulting in a value of 10E3. In addition, the number of hidden layer neurons was fixed at 500.

After verifying that the non-linear model had greater precision than the linear model, its parameters were interpreted by applying the above sensitivity analysis. The first-order analysis qualifies the contribution to the output of the different input variables (job demands, skill discretion, decision authority, supervisor support and co-worker support) in a direct way without interactions. Table 1 presents the contribution to the variance of each of these variables and their signs (which were estimated empirically).

The variables that contribute most significantly to the explanation of perceived occupational health of accountants are job demands, with a negative sign, and supervisor support, with a positive sign. These results highlight, on the one hand, that an accountant’s health deteriorates as job demands increase, while having the support of immediate superiors contributes positively to perceived occupational health. Since the results of estimation 1 and 2 are close (see Table 1), they can be considered robust. Table 1 also shows that the direct effect of the other variables (skill discretion, decision authority and co-worker support) is irrelevant in the perception of accountants’ health.

Then, we analyzed the possible moderating effect of job control and social support in the relationship between job demands and perceived occupational health. This was solved by incorporating the contribution to the variance of the different interactions of the variable two by two. 

The results of such interactions, all of them with positive signs, are presented in Table 2. The positive sign of the interactions when job demands had a negative relationship with perceived occupational health confirms the moderating effect that show both social support and job control. Focusing on Table 2, we can appreciate that the possibility of practicing skills as well as having the confidence in support from the top are the two variables that largely reduce the negative effect of job demands in perceived occupational health by the accountants. 

## 5. Discussion

Accountants play an important role in market economies. Financial information reduces costs of transaction in agency relationship; therefore, accountants are an essential link in the relations between owners and managers [70]. In this context, professional accountants that audit give credibility to the system, constituting an effective signaling mechanism in corporate governance [71] and influence in credit decisions [72]. Likewise, Positive Accounting Theory has shown the influence of accounting figures in taxation, sectoral regulation and executive compensation plans [73].

The significance and relevance of this profession places stress on accountants, who are forced to contend with strong demands that can affect their health. The most significant include professionals who work with tight deadlines, with significant seasonality (since most companies issue financial reports at the same time, coinciding with the calendar year) and in an environment of extreme competition, which reduces prices and increases pressure on resource allocation. 

In this context of strain, the accountant must “juggle” to put into practice the independence, judgement, and professional skepticism that international auditing agencies require. Therefore, any deterioration in the health of these professionals, which alter their emotional balance, will decisively affect the judgement of the professional accountant and the quality of their work.

The present paper is relevant in the European context and investigates the effects of job demands in occupational health perceived by the accountants, as well as the moderator role that job control exerts over work and the social support of colleagues and superiors. The results, after the application of a neural network model, confirm some of the hypotheses raised in this research, both with regard to the outcomes and the effects of the moderators.

On the one hand, in relation to the direct effects of the job demands, job control and social support on perceived occupational health (H1 to H5), it has been shown that job demands are the factor that best explains the deterioration of accountants’ health, which is in line with the prior literature that has verified the existence of this relationship in other manifestations of loss of health, such as burnout [38]. The other variable that exerts an important influence on perceived occupational health is supervisor support—in this case, in a positive sense. This result is consistent with the model of an organization staffed by professional accountants and is based mainly on professional talent. The implementation of professional talent requires the support and trust of the organization. The results of this study highlight better occupational health among professionals who receive explicit support from their superiors. On the other hand, job control does not exert a significant direct effect on perceived occupational health, possibly because the work of the accountants is structured and formalized, leaving little room for individual initiative.

Otherwise, the JDCS model discusses a number of mechanisms that contribute to cushioning the pernicious effect of the job demands on perceived occupational health. The results derived from the neural network model suggest that these mechanisms indeed improve the perception of one’s own occupational health—specifically, the implementation of vocational skills and perception of greater support of hierarchical superiors. Accounting firms are characterized by their high level of hierarchy and fierce pyramidal structure. This design requires a high degree of staff turnover, which allows the channels of promotion to be open [74]. Since accountants’ immediate superiors evaluate them, support from superiors is one of the mechanisms for recognition with greater impact on the development of a healthy work environment [41]. In a qualitative study, the importance of superiors in the self-esteem of subordinates is underlined: “To hear my senior say I’ve done a good job is a real boost to my morale!”. The comment of this assistant illustrates the process governing her approach to auditing: She was congratulated for her work, and she takes the compliment personally (“I’m doing a good job”), thus strengthening her identity, and this prospect is precisely what motivates her to do her best [31]. To fulfil their expectations in terms of self-achievement, some auditors go further. Undoubtedly, support of one’s superiors and a feeling of justice decrease the feeling of burnout among accountants [19,22]. In addition, the confidence of being supported by the organization and its managers in a competitive environment where lawsuits are frequent contributes to a vital balance [18].

The other variable with a significant moderating effect in the relationship between job demand and perceived occupational health is the possibility of practicing personal skills (skill discretion). The analysis of this effect, which has much to do with the concept of hardiness, is especially relevant in the profession discussed in this research. People with hardy personalities perceive stimulating and challenging situations as stressful (commitment dimension), probably because they believe that stress factors are controllable thanks to their professional skills (skill discretion), thus transforming risk into an opportunity for personal growth (challenge dimension). Commitment and challenge show a significant relationship with exhaustion, one of the fundamental dimensions of burnout, as point out Reference [26]. This result offers the possibility of aligning the needs of firms to respond to a competitive market with the professional interests of the accountants to the point where many professionals consider joining these companies as a promising start to a career or as a highly stimulating professional activity [47]. 

**Figure 3:**
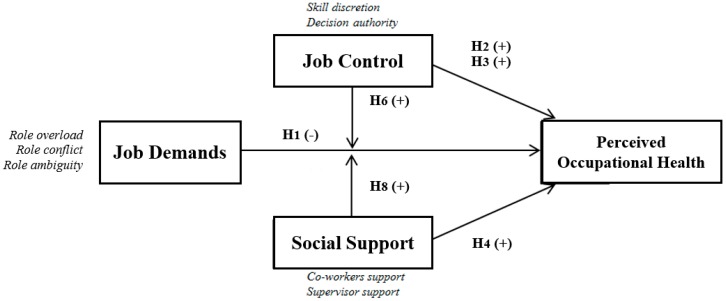
Model hypotheses confirmed.

In short (see Figure 3), the direct effects on perceived occupational health come primarily from job demands (Hypothesis 1) and the recognition of superiors (Hypothesis 4), while the incidence of the two dimensions of job control (Hypothesis 2 and 3) and co-worker support (Hypothesis 5) is not relevant. For the moderating effects, the relationship between job demand and perceived occupational health is reducing among professionals who can practice their professional skills (Hypothesis 6) and have the support of their superiors (Hypothesis 8). The rest of the moderator effects scarcely explain the variance of the model: Decision authority (Hypothesis 7) and co-worker support (Hypothesis 9).

Two important practical implications for organizations and their human resource managers can be drawn from this research. On the one hand, recruitment and selection processes should pay special attention to candidates with hardiness, i.e., those who are capable of transforming stressful situations into opportunities for growth. Professionals who have this competence profile better bear the pressure inherent in the work, and hardiness is especially valuable for developing professional careers with long spans, such as accountancy firms. On the other hand, in an environment that is as demanding as the object of this paper in which one is subjected to intense pressures of time and deadlines, superiors play a central role in the promotion of a healthy work environment. Therefore, superiors should receive practical training in managing people so that they are aware of and develop the skills needed to provide the social support that accounting professionals need and demand. Leading practices and training programmes implemented by large accountancy firms to support the mental health of employees are displayed by Reference [13].

As noted in the introduction to this research, Total Worker Health promotes integral intervention in health that considers both the individual perspective and the organizational context. In this sense, JDCS model is one of the theoretical approaches more suited to understanding and interpreting the relationships between work features and the health and well-being of employees [8]. As a conclusion, we can say that this research confirms the basic postulates of the JDCS model (professional development and supervisor support mainly constitute the basic mechanisms for moderating the high pressures of the accounting work), thus contributes to explaining the perceived occupational health of accounting profession from an original and novel theoretical framework on this field.

## 6. Limitations and Future Lines of Research

Finally, it is necessary to mention the major methodological limitations of this study. First, perceived occupational health was measured through individual self-perceptions. Second, the problem of social desirability is a setback of studies that ask about how labor conditions affect employees. Self-perception and the social desirability may cause bias in responses. Third, the relationship between the variables being investigated cannot be considered causally since we studied cross-sectional data, not experimental data. Finally, the study is limited to the scope of the European Union. Future studies should investigate the influence of the JDCS model in professional accountants who develop their activities in other cultural contexts.

## 7. Conclusions

Scarce studies have explored the main effects and multiplicative model in relation to the perceived occupational health of professional accountants. The Job Demand-Control and Job Demand-Control-Support (JDCS) models adopted in this article are an appropriate theoretical framework to analyze the relationship between the characteristics of labor and occupational health.

In conclusion, the obtained results confirm the relationship between certain stress factors that affect the health of the accountants, as well as the direct effect that has the recognition of superiors in occupational health. Additionally, the results highlight the moderating effect of professional development and the support of superiors on the job’s demands.

The implications of these findings could assist human resource managers in facilitating, to some extent, good social relationships amongst accountants.

## Figures and Tables

**Table 1:** Analysis of the first order. Contribution to the variance and sign.

	JD	SD	DA	SS	WS
Estimate 1	0.610426 (−)	0.0255825 (+)	0.0148224 (+)	0.283876 (+)	0.0231483 (+)
Estimate 2	0.560802 (−)	0.0019926 (+)	0.0151903 (+)	0.332172 (+)	0.0015737 (+)

JD (Job Demands), SD (Skill Discretion), DA (Decision Authority), SS (Social Support), WS (Co-Worker Support).

**Table 2:** Analysis of the iterations of the variables. Contribution to the variance.

	JD	SD	DA	SS	WS
JD	-	0.0543336	0.0065206	0.0761218	0.0044644
SD	**0.0847528**	-	0.0097692	0.0191648	0.0254918
DA	0.0058920	0.0104552	-	0.0004655	0.0059129
SS	**0.0800194**	0.0189461	0.0009225	-	0.0007901
WS	0.0036139	**0.0249092**	0.0056966	0.0000172	-

JD (Job Demands), SD (Skill Discretion), DA (Decision Authority), SS (Social Support), WS (Co-Worker Support). Above, the axis values correspond to interactions in Scenario 1, while values below the axis correspond to Scenario 2. In bold, the highest values are highlighted.
